# Pre-diagnostic plasma enterolactone concentrations are associated with lower mortality among individuals with type 2 diabetes: a case-cohort study in the Danish Diet, Cancer and Health cohort

**DOI:** 10.1007/s00125-019-4854-9

**Published:** 2019-04-08

**Authors:** Anne K. Eriksen, Cecilie Kyrø, Natalja P. Nørskov, Kirsten Frederiksen, Knud-Erik Bach Knudsen, Kim Overvad, Rikard Landberg, Anne Tjønneland, Anja Olsen

**Affiliations:** 1 Unit of Diet, Genes and Environment, Danish Cancer Society Research Center, Strandboulevarden 49, 2100 Copenhagen, Denmark; 2 Department of Animal Science, Aarhus University, Tjele, Denmark; 3 Unit of Statistics and Pharmacoepidemiology, Danish Cancer Society Research Center, Copenhagen, Denmark; 4 Department of Public Health, Section for Epidemiology, Aarhus University, Aarhus C, Denmark; 5 Department of Biology and Biological Engineering, Chalmers University of Technology, 412 96 Gothenburg, Sweden; 6 Department of Public Health, University of Copenhagen, Copenhagen, Denmark

**Keywords:** Case-cohort, Enterolactone, Mortality, Prognosis, Type 2 diabetes

## Abstract

**Aims/hypothesis:**

The phytoestrogen enterolactone is a gut microbiota-derived metabolite of plant lignans with suggested beneficial properties for health. In the current study, we investigated the association between pre-diagnostic plasma enterolactone concentrations and mortality among individuals diagnosed with type 2 diabetes.

**Methods:**

In a population of people diagnosed with diabetes, nested within the Danish Diet, Cancer and Health cohort, we conducted a case-cohort study including a random sample of *n* = 450 cases (deceased) and a randomly selected subcohort of *n* = 850 (in total *n* = 617 deaths). Information on diagnosis, vital status and cause of death was obtained from Danish registers. Cox proportional hazard models with special weighting were applied to assess all-cause and cause-specific mortality.

**Results:**

The median enterolactone concentration of the current population was low, 10.9 nmol/l (5th percentile to 95th percentile: 1.3–59.6), compared with previously reported concentrations from the Diet, Cancer and Health cohort. Pre-diagnostic enterolactone concentrations were associated with lower all-cause mortality when assessed linearly per doubling in concentration (log_2_) (HR 0.91 [95% CI 0.85, 0.96]) and according to quartiles (HR 0.63 [95% CI 0.48, 0.84]) for the highest quartile of enterolactone compared with the lowest quartile. For cause-specific mortality, only death from diabetes (registered as underlying cause of death) reached statistical significance.

**Conclusions/interpretation:**

Based on this large cohort of people with diabetes with detailed and complete baseline and follow-up information, pre-diagnostic enterolactone concentrations were inversely associated with mortality. To our knowledge, this is the first study on enterolactone and type 2 diabetes mortality. Our findings call for further exploration of enterolactone in type 2 diabetes management.

**Electronic supplementary material:**

The online version of this article (10.1007/s00125-019-4854-9) contains peer-reviewed but unedited supplementary material, which is available to authorised users.

## Introduction

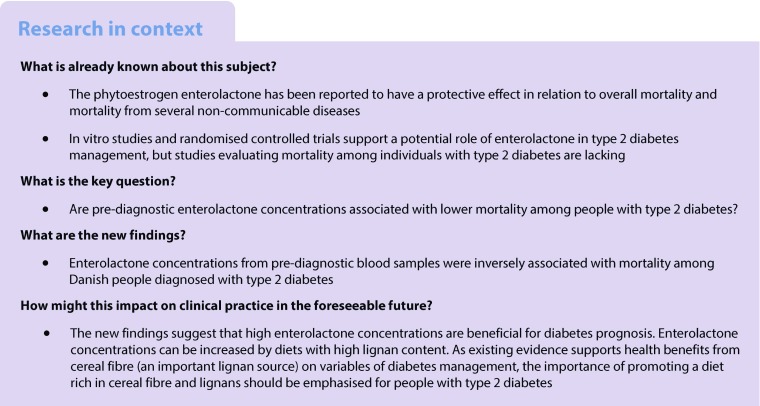



Dietary factors, such as whole-grain intake, have been related to a lower mortality from type 2 diabetes [1], and soluble fibre has been shown to reduce HbA_1c_ and fasting plasma glucose in individuals diagnosed with type 2 diabetes [2]. Furthermore, among people with type 2 diabetes, high LDL-cholesterol has been associated with higher risk of cardiovascular mortality, which is a common cause of premature death in type 2 diabetes [3]. Although further investigations are required, dietary interventions with whole-grain rye [4] and flaxseed [5] have indicated beneficial effects on LDL-cholesterol. Thus, diet plays a role in the management of type 2 diabetes and good management may improve survival rate.

Enterolactone, a phytoestrogen metabolite produced by the gut microbiota after ingestion of dietary lignans, has been studied widely for its protective effects in chronic disease [6]. The main dietary sources include seeds, whole grains, nuts and fibre-rich fruits and vegetables, with smaller amounts found in coffee, tea, wine and beer [7]. The metabolism of lignans into enterolignans (enterodiol and enterolactone) is affected by several factors including smoking, obesity, dietary sources of lignans [6], gut microbiota [8] and use of antibiotics [9].

The role of enterolactone in type 2 diabetes management remains unstudied in observational investigations but intervention studies suggest favourable associations between lignan intake and several factors associated with management of type 2 diabetes: glycaemic control, inflammatory markers and cholesterols [10–12]. The observed associations related to factors of importance for diabetes management call for further investigation into whether enterolactone may be a protective factor for mortality among people with diabetes. Therefore, the aim of the present study was to investigate prospectively the association between pre-diagnostic plasma enterolactone concentrations and mortality rate ratio among people diagnosed with type 2 diabetes in the Diet, Cancer and Health cohort [13]. The association was examined for all-cause mortality as the primary outcome and secondarily for cause-specific mortality. The hypothesis was that high plasma enterolactone concentrations are associated with lower mortality risk (rate ratio) compared with low concentrations.

## Methods

### Study population and follow-up

The Danish Diet, Cancer and Health cohort is a prospective cohort study [13]. At baseline (between 1993 and 1997), men and women aged 50–64 years and living in the counties of Copenhagen and Aarhus were invited to participate, which 36% (*n* = 57,053) agreed to. At the baseline visit, participants completed lifestyle- and dietary questionnaires [14] and trained personnel conducted physical examinations and blood sampling. The blood samples (30 ml) were processed and frozen in liquid nitrogen vapour (−150°C) within 2 h.

The 57,053 cohort participants were followed for incident diabetes in the Danish Diabetes Registry from baseline until the end of 2009 [15]. Inclusion in the register was based on the following diagnostic criteria: (1) registration in the National Patient Register with a diagnosis of diabetes; (2) registration of chiropody (as diabetic patient) in the Danish National Health Service Register (NHSR); (3) five blood-glucose measurements in a 1 year period in the Danish NHSR; (4) two blood-glucose measurements per year for five consecutive years in NHSR; (5) purchase of oral glucose-lowering drugs in the Danish National Prescription Registry (DNPR) [16] and (6) purchase of prescribed insulin recorded in DNPR. As criteria 3 and 4 have the lowest positive predictive values [15], cohort participants registered with one of these criteria alone were not defined as ‘cases’. The number of incident diabetes cases was 6177. Of these, 1526 were diagnosed based on criteria 3 or 4 only and therefore not included; an additional 168 cases were excluded due to a lack of plasma sample, resulting in 4483 eligible people with diabetes. It is not possible to distinguish between type 1 diabetes and type 2 diabetes cases based on the information available in the Danish Diabetes Registry [15]. However, as the baseline age was 50+ and the median age at diagnosis 65 years, we assume that the vast majority were diagnosed with type 2 diabetes and hereafter refer to diabetes as type 2 diabetes.

Vital status was obtained through linkage to The Danish Civil Registration System [17] and cause-specific death was obtained from the Danish Register of Causes of Death [18]. Causes of death were grouped by the following ICD-10 code categories (http://apps.who.int/classifications/icd10/browse/2016/en): cardiovascular diseases (I-); cancer (C-); diabetes (E10-14); respiratory diseases (J-) and other causes (including infections) (A-, B-), accidents/suicides/assaults (V-, W-, X-, Y-), renal failure (N-), mental disorders (F-), nervous system disorders (G-), digestive disorders (K-), other endocrine disorders (E-) and musculoskeletal diseases (M-). Mortality rate ratio from all causes was the primary endpoint and cause-specific mortality outcomes were secondary. For the present study, information on vital status was available until 1 February 2016 whereas information on cause of death only could be obtained until 31 December 2015. This leaves 13 deaths only included in the analyses of all-cause mortality.

In total, 1402 people among the population of 4483 diagnosed with type 2 diabetes died during the follow-up period from date of diagnoses (before 31 December 2009) and end of follow-up on 1 February 2016.

For the current study, a case-cohort design was applied [19]. A random subcohort of 850 individuals was sampled from the 4483 people in the study population. Of these, 268 died during follow-up. Further, *n* = 450 cases from the 1402 deceased individuals with type 2 diabetes were randomly sampled. By coincidence, 101 people with type 2 diabetes were already part of the subcohort. Therefore, our study base consisted of a subcohort of 850 individuals, including 268 deaths, and an additional 349 cases (450 minus the 101 individuals overlapping) included in the study according to the principles of the case-cohort design (*n* = 617 deaths). Following exclusions (missing information on covariates, no available sample or failure enterolactone measurement), the final subcohort consisted of 841 individuals and there were 610 deaths (268 within the subcohort + 342 additional cases). Causes of death covered diabetes-specific death where diabetes as such was registered as the underlying cause of death (*n* = 48), cardiovascular diseases (*n* = 141), cancer (*n* = 243), respiratory diseases (*n* = 63), other diseases (*n* = 102) or unknown causes (*n* = 13).

### Laboratory analyses

Enterolactone concentrations were determined using a liquid chromatography–tandem mass spectrometry (LC-MS/MS) method [20] wherein the intact form of enterolactone was measured as glucuronide-conjugated, sulphate-conjugated or free enterolactone. The sum of these three forms constitutes the total enterolactone concentration. Robustness of the enterolactone assay was validated both during method development [20] and during sample analyses using six quality-control samples in each batch. During method development, inter-batch variation was calculated to be lower than 10%, which was similar during sample analyses.

### Health score

Because general health is a strong predictor of mortality [21], we wanted to explore whether the association between enterolactone and mortality could be explained by enterolactone being a marker of general health. Therefore, we created a score based on three health behaviours. BMI, smoking status and physical (in)activity are known mortality risk factors [21] and were chosen to best reflect general health status based on the available baseline questionnaire information. We expected that any association between enterolactone and mortality would disappear in the health-score-stratified analysis if enterolactone level was simply an indicator of general health. The three factors were scored from 0 to 2 points, with the highest score representing the healthiest behaviour as follows: BMI ≤27 kg/m^2^ (score 2), 27–32 kg/m^2^ (score 1), >32 kg/m^2^ (score 0); smoking status never (score 1), former (score 0.5), current (score 0); participation in sport activities yes (score 1), no (score 0). The total score ranged from 0 to 4 points and was further divided into a low (≤1.5), medium (1.5–3.0) and high (≥3) category of healthy lifestyle.

### Information on antibiotics

Use of antibiotics is known to affect enterolactone concentrations [22]. Therefore, information about redeemed prescriptions for systemic antibiotics, within 12 months prior to baseline, was obtained from the DNPR [16]. Antibiotic use was categorised in three groups based on the most recent filling of prescriptions: 0–3 months before baseline; 3–12 months before baseline or no use (0–12 months before baseline). As the registry was established in 1995, only participants with baseline from 1 January 1996 and onwards could be registered in the database with sufficient information to go 12 months back (*n* = 596, 50% of total).

### Ethics

The study was approved by the regional ethical committees on human studies in Copenhagen and Aarhus [File (KF)11-037/01] and by the Danish Data Protection Agency. All study participants gave written informed consent. The Danish authorities (Sundhedsdatastyrelsen) approved linkage with the DNPR and these analyses were conducted through Statistics Denmark.

### Statistical methods

The association between plasma enterolactone concentrations and mortality was assessed in a case-cohort design by Cox proportional hazard models with cases outside the subcohort entering the risk set just before their event time as suggested by Prentice [19] using robust variance estimation [23]. The subcohort members contributed with follow-up time from date of diagnosis to date of exit (death, emigration or end of follow-up at 1 February 2016 for all-cause mortality and at 31 December 2015 for cause-specific mortality) and time since diagnosis was used as the underlying time scale. Initially, age in 5 year bands and sex was included in strata to allow for different underlying hazards. Linearity of the dose–response association for the main exposure, plasma enterolactone concentration and the continuous potential confounders were tested by linear splines. According to this, enterolactone was log_2_ transformed to obtain a linear association and BMI showing a U-shaped association was categorised into three groups (≤27 kg/m^2^, 27–32 kg/m^2^, >32 kg/m^2^). The remaining continuous potential confounders (alcohol intake, time between baseline and diagnosis, blood pressure, number of hours spent participating in sports, bowel movements, smoking duration and intensity and consumption of sugar-sweetened beverages, red meat and processed meat) showed no deviations from linearity.

The proportional hazards assumption was assessed for the main exposure variable and the confounders by correlation tests of the Schoenfeld residuals as proposed by Xue et al [24]. Smoking violated the proportional hazards assumption (all *p* < 0.003) but after including it in strata together with age and sex, none of the remaining variables violated the proportional hazards assumption (all *p* ≥ 0.25).

Model 1, the crude model, was stratified in 5 year age bands and by sex. Model 2 was additionally adjusted for smoking and BMI, as both were considered important risk factors and thus included a priori [21, 25]. In model 3, additional potential confounders were included based on an approach of >10% change in exposure coefficient estimate [26]. Based on this (>10%), sports participation (yes/no) and alcohol intake (alcohol abstain yes/no, intake among users, linear) were included. Furthermore, time between baseline and diagnosis (linear), blood pressure (systolic and diastolic, mmHg), time spent participating in sports (h/week), bowel movements (no. of times/week), additional information on tobacco use (smoking duration and intensity, no. of cigarettes per day or year), consumption of sugar-sweetened beverages (g/day), red meat (g/day) and processed meat (g/day), number of years of schooling (≤7 years, 8–10 years, ≥11 years), hormone use (never, former, current, male sex hormones) and menopausal status (postmenopause/premenopause) were tested as potential confounders but were not included (change in β-estimate <10%). No effect modification by age (*p* = 0.77), sex (*p* = 0.13) or menopausal status (*p* = 0.17) was observed.

### Sensitivity analyses

To investigate whether the association between enterolactone concentration and all-cause mortality was consistent across subgroups according to general health status, we estimated the linear association between enterolactone and mortality within each of the three health-score categories (low, middle, high) in one model. Effect modification by health-score category was tested by combining health-score category with quartile of enterolactone [27]. No interaction between health-score category and enterolactone quartile was observed (*p* = 0.77).

Further, a sensitivity analysis was conducted to investigate whether use of antibiotic medication affected the association between enterolactone concentration and mortality. This was done by adding an antibiotic variable (0–3 months before baseline, 3–12 months before baseline or no use [12 months preceding baseline]) to the adjusted model for the subset of participants (*n* = 596) for which information on antibiotic medication was available. A separate analysis among non-users of antibiotics (*n* = 430) was likewise conducted.

The statistical analyses were conducted in SAS statistical software release 9.3 (SAS Institute, Cary, NC, USA).

## Results

The case-cohort study population included 1183 individuals diagnosed with type 2 diabetes, a randomly selected subcohort of 841, and 342 deceased cases in the sampled deceased group. The median age at type 2 diabetes diagnosis was 65 years (5th to 95th percentile 55–74), which was 7.4 years (5th to 95th percentile 0.4–13.4) after blood sampling. Sixty per cent were men and the median follow-up time was 10.7 years for the subcohort. During the follow-up time, 268 people from the subcohort died, resulting in 610 deaths when combining deaths in the subcohort and the sampled deceased group.

Among the subcohort of people diagnosed with type 2 diabetes, 60% were men, the median age at diagnosis was 64 years (5th to 95th percentile 54–74) and the follow-up time for all-cause mortality was 10.7 years (5th to 95th percentile 1.8–18.5). At study baseline, median BMI was 28.8 kg/m^2^, the proportion of current smokers was 41% and 43% engaged in sports activity (Table 1). The entire Diet, Cancer and Health cohort had a lower proportion of men (47%), current smokers (36%), a lower median BMI (25.5 kg/m^2^) and a higher participation in sports (54%) [13] compared with the sub-group of people with diabetes included in the current study. Median enterolactone concentrations measured for previously published studies (*n* = 2237) were 21.3 nmol/l for women and 18.6 nmol/l for men [9]. Pre-diagnostic baseline characteristics are presented by quartile of enterolactone concentrations for the subcohort only (Table 2), and according to group (subcohort and deceased). The deceased group was further subdivided according to cause of death (Table 1).

**Table 1:** Baseline characteristics by subcohort, and deceased by cause-specific mortality: a case-cohort study in the Diet, Cancer and Health cohort (*n* = 1183)

Characteristic	Subcohort	Deceased					
	All *n* = 841	All *n* = 610	Diabetes *n* = 48	Cardiovascular *n* = 141	Cancer *n* = 243	Respiratory *n* = 63	Other *n* = 102
Age at diagnosis, years	64 (54–74)	65 (55–75)	63 (53–74)	65 (56–75)	65 (56–74)	65 (57–73)	65 (54–76)
Follow-up time^a^, years	10.7 (1.8–18.5)	7.1 (0.2–15.7)	11.3 (0.8–18.4)	6.2 (0.08–13.8)	6.6 (0.1–15.0)	9.4 (2.6–17.0)	8.1 (0.3–15.1)
Pre-diagnostic (at study baseline)							
Enterolactone, nmol/l	10.9 (1.3–59.6)	9.5 (1.1–49.7)	6.5 (1.3–22.1)	11.0 (1.2–43.4)	8.9 (1.1–49.8)	9.4 (1.3–62.5)	9.1 (1.1–43.2)
Male sex, *n* (%)	508 (60)	413 (68)	28 (58)	102 (72)	166 (68)	38 (60)	70 (69)
BMI, kg/m^2^	28.8 (22.8–37.6)	28.8 (22.1–40.8)	29.8 (24.2–41.5)	29.0 (23.4–42.1)	28.8 (21.9–37.8)	27.6 (20.1–38.2)	28.1 (21.8–36.1)
Height, cm	172 (157–184.5)	172 (157–184)	171 (154–184)	173 (158–184)	173 (157–185)	169 (156–183)	171 (160–186)
Waist circumference, cm	99 (77–121)	100 (77–126)	102 (83–130)	100 (83–131)	101 (77–122)	100 (72–123)	99 (76–125)
Systolic blood pressure, mmHg	146 (117–184)	151 (119–190)	160 (116–189)	155 (121–201)	147 (119–189)	147 (120–184)	152 (117–184)
Diastolic blood pressure, mmHg	87 (69–105)	88 (70–108)	90 (72–111)	89 (72–111)	88 (70–106)	86 (69–105)	89 (68–108)
Serum cholesterol, mmol/l	6.1 (4.3–8.2)	6.1 (4.2–8.5)	6.0 (3.9–8.5)	6.5 (4.3–8.7)	6.1 (4.3–8.3)	5.7 (3.8–7.6)	6.0 (4.4–8.1)
Whole-grain intake, g/day	37.6 (9.0–79.3)	37.2 (7.4–75.7)	38.0 (9.3–71.7)	39.3 (11.0–78.3)	37.1 (7.4–75.7)	37.7 (10.8–77.0)	31.0 (5.6–70.7)
Cabbage intake, g/day	13.2 (2.0–42.6)	11.1 (1.7–37.5)	14.4 (2.8–45.0)	10.7 (2.0–31.0)	11.0 (1.6–38.6)	10.1 (1.8–36.6)	11.2 (1.8–37.1)
Leafy vegetable intake, g/day	6.1 (0.5–34.0)	4.2 (0–26.4)	4.2 (0.5–35.1)	4.2 (0–22.6)	4.1 (0.3–34.7)	4.8 (0.4–26.0)	4.2 (0–25.5)
Red meat intake, g/day	88 (39–178)	87 (39–191)	83 (49–169)	88 (39–191)	88 (38–216)	81 (34–178)	87 (41–168)
Processed meat intake, g/day	30 (7–81)	34 (8–96)	35 (7–101)	34 (8–131)	35 (8–93)	28 (7–91)	32 (8–89)
Coffee consumption, no. of cups/day	4.5 (0.1–8)	4.5 (0–8)	4.5 (0–8)	4.5 (0.4–8)	4.5 (0–8)	4.5 (0–8)	4.5 (0–8)
Sugar-sweetened beverage consumption, g/day	20 (0–516)	20 (0–529)	16 (0–500)	29 (0–900)	20 (0–503)	32 (0–700)	20 (0–529)
Alcohol intake among users, g/day	13.7 (0.8–69.4)	16.1 (0.7–84.7)	11.8 (0.4–57.3)	14.3 (0.7–81.5)	16.0 (1.0–85.3)	12.6 (0.4–83.9)	20.3 (1.0–114.5)
Alcohol abstainers, *n* (%)	26 (3)	26 (4)	3 (6)	7 (5)	8 (3)	4 (6)	4 (4)
Bowel movements/week	4 (2–5)	4 (2–5)	4 (2–6)	4 (2–6)	4 (3–5)	4 (2–5)	4 (3–5)
Sport (yes), *n* (%)	359 (43)	186 (31)	13 (27)	43 (31)	71 (29)	14 (22)	38 (37)
Sport, h/week	1.5 (0.5–6.0)	2.0 (0.5–7.0)	1.5 (0.5–5.0)	1.5 (0.5–5.0)	2.0 (0.5–6.0)	1.5 (0.5–18.0)	2.0 (0.5–10.0)
School, *n* (%)							
Low	351 (42)	299 (49)	22 (46)	74 (52)	106 (44)	38 (60)	53 (52)
Middle	372 (44)	247 (41)	21 (44)	46 (33)	115 (47)	22 (35)	36 (35)
High	118 (14)	64 (11)	5 (10)	21 (15)	22 (9)	3 (5)	13 (13)
Smoking status, *n* (%)							
Never	230 (27)	122 (20)	11 (23)	27 (19)	50 (21)	8 (13)	23 (23)
Former	267 (32)	158 (26)	11 (23)	48 (34)	59 (24)	10 (16)	25 (25)
Current	344 (41)	330 (54)	26 (54)	66 (47)	134 (55)	45 (71)	54 (53)
Former and current smokers							
Years of smoking, years	33 (8–47)	37 (10–48)	37 (15–46)	38 (3–49)	37 (10–47)	40 (23–48)	36 (10–48)
Pack-years, years	25.6 (2.5–60.8)	30 (3.5–67)	27.5 (5.8–51.5)	28.0 (1.5–82.9)	31 (3.5–71.0)	30.5 (12.5–76.5)	29.5 (1.5–55.8)
Current smokers							
Tobacco, g/day	20 (5–67)	20 (5–40)	20 (5–40)	20 (5–37)	20 (5–44)	20 (10–34)	20 (3–30)
Menopausal hormone therapy in women, *n* (%)							
Never	190 (57)	106 (54)	12 (60)	23 (59)	42 (55)	13 (52)	14 (44)
Former	62 (19)	43 (22)	6 (30)	7 (18)	16 (21)	4 (16)	9 (28)
Current	81 (24)	48 (24)	2 (10)	9 (23)	19 (25)	8 (32)	9 (28)
Menopausal state of women (post), *n* (%)	284 (85)	176 (89)	18 (90)	35 (90)	67 (87)	23 (92)	29 (91)
0–12 months before study baseline							
Antibiotic medication, *n* (%)							
Missing data (baseline before 1996)	389 (46)	336 (55)	27 (56)	90 (64)	113 (47)	40 (63)	60 (59)
Among the subgroup with information (baseline 1996 and onwards), *n* (%)	452	274	21	51	130	23	42
No use (12 months preceding baseline)	328 (73)	193 (70)	15 (71)	38 (75)	89 (68)	15 (65)	31 (74)
0–3 months before baseline	43 (10)	32 (12)	6 (29)^b^	13 (25)^b^	41 (32)^b^	8 (35)^b^	11 (26)^b^
3–12 months before baseline	81 (18)	49 (18)					
Baseline enterolactone, nmol/l							
No antibiotic use (12 months before baseline)	11.8 (1.5–59.8)	10.2 (1.2–46.0)	6.7 (1.6–24.3)	11.0 (0.6–39.6)	9.9 (1.2–48.5)	13.8 (2.7–47.5)	12.2 (1.3–29.8)
Antibiotic use (0–12 months before baseline)	8.9 (1.0–59.2)	10.1 (1.0–67.0)	8.7 (2.1–11.3)	9.9 (0.9–34.1)	10.6 (0.8–82.4)	9.6 (2.4–32.0)	6.6 (1.9–67.0)

Data are shown as median (5th–95th percentile) or *n* (%)

^a^Time between diagnosis and exit, only subcohort (*n* = 841)

^b^Due to the low number of participants, the groups 0–3 and 3–12 months before baseline were grouped together

**Table 2:** Baseline characteristics of subcohort by quartile of enterolactone concentration of individuals with type 2 diabetes: a case-cohort study in the Diet, Cancer and Health cohort (*n* = 841)

Characteristic	1st quartile (0–5.42 nmol/l) *n* = 210	2nd quartile (5.45–10.89 nmol/l) *n* = 210	3rd quartile (10.92–22.54 nmol/l) *n* = 211	4th quartile (22.88–296.30 nmol/l) *n* = 210
Age at diagnosis, years	64 (54–73)	65 (54–75)	64 (55–74)	64 (55–74)
Follow-up time^a^, years	10.5 (2.4–18.5)	10.5 (0.7–18.1)	10.7 (2.2–18.5)	10.7 (2.3–18.7)
Deceased, *n* (%)	83 (40)	67 (32)	61 (29)	57 (27)
Pre-diagnostic (at study baseline)				
Enterolactone concentration, nmol/l	2.92 (0.64–5.14)	7.75 (5.66–10.28)	15.78 (11.26–21.91)	38.29 (23.90–110.53)
Male sex, *n* (%)	131 (62)	145 (69)	123 (58)	109 (52)
BMI, kg/m^2^	29.3 (22.8–37.6)	28.6 (23.3–37.5)	29.4 (23.9–37.5)	28.4 (22.1–37.8)
Height, cm	172 (159–184)	172 (157–184)	172 (156–186)	171 (157–186)
Waist circumference, cm	100 (78–123)	100 (77–120)	100 (77–121)	96 (75–120)
Systolic blood pressure, mmHg	148 (117–186)	146 (119–182)	146 (119–185)	146 (112–183)
Diastolic blood pressure, mmHg	88 (70–118)	87 (72–104)	87 (68–105)	87 (69–103)
Serum cholesterol, mmol/l	6.0 (4.0–8.3)	6.2 (4.5–8.2)	6.1 (4.3–8.3)	6.2 (4.4–8.1)
Whole-grain intake, g/day	31.7 (6.7–79.4)	37.8 (9.4–74.3)	38.2 (11.2–81.3)	39.5 (17.1–80.9)
Cabbage intake, g/day	10.4 (1.6–38.6)	11.7 (2.3–40.5)	15.7 (3.0–41.3)	14.9 (1.9–46.4)
Leafy vegetable intake, g/day	4.0 (0.4–33.8)	4.7 (0.2–29.9)	7.6 (1.2–36.6)	7.4 (0.6–36.0)
Red meat intake, g/day	93 (42–184)	90 (39–192)	90 (36–166)	82 (35–169)
Processed meat intake, g/day	32 (8–93)	33 (7–85)	29 (7–85)	25 (6–69)
Coffee consumption, no. of cups/day	4.5 (0–8)	4.5 (0.4–8)	4.5 (0.1–8)	4.5 (0–8)
Sugar-sweetened beverage consumption, g/day	29 (0–516)	30 (0–516)	20 (0–286)	16 (0–503)
Bowel movements/week	4 (3–6)	4 (2–5)	4 (2–5)	4 (2–5)
Alcohol intake, g/day	15.3 (1.0–80.3)	14.8 (0.6–68.9)	14.7 (1.2–67.5)	12.4 (0.8–63.6)
Abstainers, *n* (%)	6 (3)	10 (5)	6 (3)	4 (2)
Sport (yes), *n* (%)	62 (30)	84 (40)	106 (50)	107 (51)
Sport, h/week	2.0 (0.5–6.0)	1.5 (0.5–6.0)	1.5 (0.5–7.0)	1.5 (0.5–4.0)
School, *n* (%)				
Low	97 (46)	85 (40)	84 (40)	85 (40)
Middle	90 (43)	95 (45)	96 (45)	91 (43)
High	23 (11)	30 (14)	31 (15)	34 (16)
Smoking status, *n* (%)				
Never	54 (26)	41 (20)	69 (33)	66 (31)
Former	50 (24)	69 (33)	71 (34)	77 (37)
Current	106 (50)	100 (48)	71 (34)	67 (32)
Former and current smokers				
Years of smoking, years	35 (12–46)	34 (8–49)	32 (6–48)	30 (9–45)
Pack-years, years	29 (5–66)	26 (3–59)	24 (2–67)	22 (1–56)
Current smokers				
Tobacco, g/day	20 (10–40)	18 (6–36)	20 (4–45)	18 (2–32)
Menopausal hormone therapy in women, *n* (%)				
Never	43 (54)	43 (66)	47 (53)	57 (56)
Former	9 (11)	11 (17)	20 (23)	22 (22)
Current	27 (34)	11 (17)	21 (24)	22 (22)
Menopausal state in women (post), *n* (%)	71 (90)	57 (88)	74 (84)	82 (81)
0–12 months before study baseline				
Antibiotic medication, *n* (%)				
Missing (baseline before 1996)	97 (46)	98 (47)	100 (47)	94 (45)
Among the subgroup with information, *n* (baseline 1996 and onwards), %	113	112	111	116
No use (12 months preceding baseline)	75 (66)	83 (74)	86 (77)	84 (72)
0–3 months and 3–12 months before baseline^b^	38 (34)	29 (26)	25 (23)	32 (28)
Baseline enterolactone, nmol/l				
No antibiotic use (12 months before baseline)	2.9 (0.7–5.2)	8.2 (5.6–10.5)	15.2 (11.6–21.6)	36.7 (24.1–96.1)
Antibiotic use (0–12 months before baseline)	2.4 (0.5–5.1)	7.0 (5.6–10.1)	15.8 (11.3–20.2)	42.0 (23.6–83.0)

Data are shown as median (5th–95th percentile) or *n* (%)

^a^Time between diagnosis and exit, only subcohort (*n* = 841)

^b^Due to the low number of participants, the groups 0–3 and 3–12 months before baseline were grouped together

Overall, the plasma enterolactone concentrations measured in blood samples from baseline were low, with a median of 10.9 nmol/l (5th to 95th percentile 1.3–59.6) in the subcohort (Table 1). The concentrations varied by cause of death, with the lowest enterolactone concentrations observed among those who died from type 2 diabetes (6.5 nmol/l, 5th to 95th percentile 1.3–22.1).

**Fig. 1:**
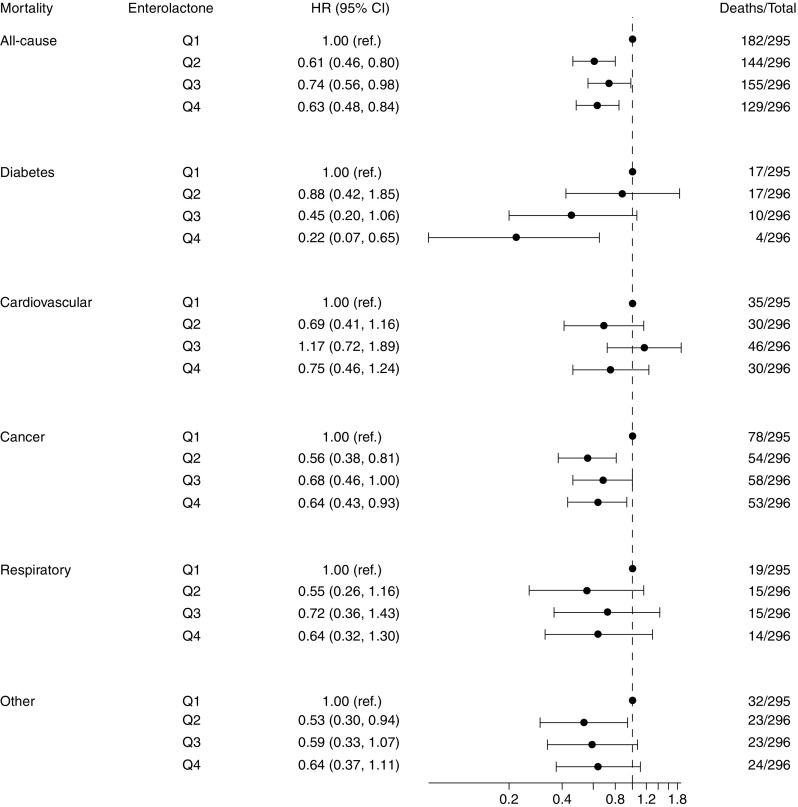
Forest plot of quartiles (Q1–Q4) of enterolactone concentration plotted on logarithmic scale (log_2_) associated with cause of death adjusted for sex, age (5 year bands), smoking and BMI among individuals with type 2 diabetes in a case-cohort study based on the Danish Diet, Cancer and Health cohort

Higher pre-diagnostic plasma enterolactone concentrations, assessed linearly per doubling in concentration (log_2_), were associated with lower all-cause mortality (HR_model2_ 0.91 [95% CI 0.85, 0.96]) (Table 3). When deaths were further subdivided according to cause, the HR_model2_ for diabetes mortality was 0.75 (95% CI 0.64, 0.86; based on 48 deaths due to diabetes). The HR_model2_ was also assessed by quartiles of enterolactone concentration; all-cause mortality was 37% lower (HR 0.63 [95% CI 0.48, 0.84]) and diabetes mortality was 78% lower (HR 0.22 [95% CI 0.07, 0.65]) for people in the highest quartile compared with those in the lowest quartile. For the remaining causes of death, results pointed towards lower mortality with higher enterolactone levels but none of the estimates reached statistical significance (Fig. 1). Additional adjustment for sports participation and alcohol intake (Model 3) revealed similar results.

**Table 3:** HR (95% CI) for pre-diagnostic plasma enterolactone concentrations and all-cause and cause-specific mortality among individuals diagnosed with type 2 diabetes: a case-cohort study based on the Diet, Cancer and Health cohort

Model	All causes *n* = 610	Diabetes *n* = 48	Cardiovascular *n* = 141	Cancer *n* = 243	Respiratory *n* = 63	Other *n* = 102
Model 1^a^	0.88 (0.83, 0.93)	0.72 (0.62, 0.83)	0.92 (0.82, 1.03)	0.88 (0.81, 0.95)	0.89 (0.77, 1.03)	0.88 (0.78, 0.99)
Model 2^b^	0.91 (0.85, 0.96)	0.75 (0.64, 0.86)	0.94 (0.84, 1.05)	0.91 (0.84, 1.00)	0.95 (0.82, 1.11)	0.90 (0.80, 1.01)
Model 3^c^	0.94 (0.88, 1.00)	0.76 (0.65, 0.88)	0.97 (0.86, 1.09)	0.95 (0.87, 1.03)	0.98 (0.84, 1.15)	0.94 (0.83, 1.07)

Data are presented as HR (95% CI), linear, by doubling in enterolactone concentration (log_2_)

^a^Adjusted for sex, age (5 year bands)

^b^Adjusted for sex, age (5 year bands), smoking (never, former, current), BMI status (<27, 27–32, >32 kg/m^2^)

^c^Adjusted for sex, age (5 year bands), smoking (never, former, current), BMI status (<27, 27–32, >32 kg/m^2^), sports participation (yes/no), alcohol intake (alcohol abstain yes/no, intake among users, linear)

If enterolactone is a marker of general health rather than being causally related, we suspected that stratifying by health status would attenuate the association between enterolactone and mortality. The inverse association observed in the stratified analysis seemed apparent across all three health-score categories (Table 4). Finally, antibiotic use did not alter the association between enterolactone and mortality in the subgroup for whom we were able to account for antibiotics (electronic supplementary material [ESM] ESM Table 1). Sensitivity analyses were performed showing similar associations in the subgroup that did not use antibiotics in the 12 months before baseline.

**Table 4:** HR (95% CI) for pre-diagnostic enterolactone concentrations and all-cause mortality by health-score category in people with type 2 diabetes: a case-cohort study based on the Diet, Cancer and Health cohort

Variable	Health score		
	Low	Middle	High
Individuals, *n*	483	443	257
Deaths, *n*	284	222	104
Quartile of enterolactone concentration			
Q1 (0–4.97 nmol/l)	1.00 (ref)	0.65 (0.42, 0.99)	0.47 (0.25, 0.87)
Q2 (4.98–10.27 nmol/l)	0.52 (0.35, 0.78)	0.49 (0.31, 0.76)	0.38 (0.22, 0.64)
Q3 (10.28–21.54 nmol/l)	0.63 (0.42, 0.95)	0.60 (0.39, 0.93)	0.35 (0.21, 0.57)
Q4 (21.55–296.30 nmol/l)	0.66 (0.43, 1.01)	0.39 (0.25, 0.60)	0.33 (0.20, 0.55)
Linear, by doubling in enterolactone concentration (log_2_)	0.92 (0.86, 0.99)	0.89 (0.82, 0.96)	0.91 (0.81, 1.02)

Adjusted for sex, age (5 year bands), alcohol intake (alcohol abstain yes/no, intake among users, linear)

## Discussion

In this population-based case-cohort study to examine the association between pre-diagnostic enterolactone concentrations and mortality among individuals diagnosed with type 2 diabetes, we found that high concentrations were associated with lower mortality. We observed a borderline significant association for all-cause mortality and markedly lower HRs for diabetes-specific mortality, although the latter was based on a limited number of cases. For the quartile estimates of all-cause mortality, there seemed to be a threshold effect whereby individuals in the second to fourth quartile had a 26–39% lower risk compared with the lowest quartile. For diabetes-specific mortality, a dose–response relationship with lower HRs by higher quartile of enterolactone was observed. Similar associations were observed across health-score categories; additional adjustments resulted in similar estimates, suggesting that enterolactone is not merely a marker of general health. There was no association between enterolactone and other cause-specific mortality outcomes.

The case-cohort design of the present study has the advantage of combining the temporal design of the large prospective study and the case−control design, allowing for an oversampling of cases (the sampled deceased group) to increase study power. Furthermore, detailed information on baseline lifestyle behaviour enabled thorough adjustment for potential confounders. Enterolactone was measured in blood samples taken before the participants were diagnosed with type 2 diabetes and thus the concentrations can be assumed to be unaffected by disease status. The metabolism of lignans into enterolignans (enterodiol and enterolactone) is affected by several factors including smoking and obesity [6]. Another important factor for the conversion of dietary lignans to enterolactone is the microbiota. The final conversion from enterodiol to enterolactone is known to be carried out by niche groups of microorganisms [28] and can be affected by antibiotic medication for over a year following administration [9]. We were able to adjust for that through linkage to the DNPR. However, the findings in the present study deviated from existing evidence (i.e. antibiotics did not affect the results). Case ascertainment, vital status and cause of death were obtained from Danish registries, which are known to be of high quality [29] as they contain nearly complete information through the personal identification number of all people living in Denmark.

The study also has some weaknesses to consider when interpreting the results. The Cause of Death Registry relies on the correctness of the physicians’ information and the coding in the National Board of Health. Autopsies are performed rarely and therefore uncertainty of the causes of deaths exists [18]. However, overall mortality was the primary outcome in this study and information on vital status was complete and unaffected by potential misclassification of underlying cause of death. Cause-specific mortality was a secondary outcome but was based solely on the correctness of the Causes of Death Registry. The median time between blood sampling and diabetes diagnosis was 7.4 years and some participants might have changed dietary habits or other lifestyle behaviours affecting enterolactone concentrations, disease risk and perhaps prognosis during this period. Our results can consequently not be used to make direct conclusions on dietary changes among people already diagnosed with diabetes. On the contrary, exposure measurements conducted close to the time of diagnosis increase the risk of reverse causality. Repeated measurements of enterolactone concentrations at different points in disease development would have been a study strength but were not conducted. Reassuringly though, repeated samples in a recent breast cancer study based on the same cohort showed good agreement between baseline and diagnostic enterolactone concentrations [30]. Another factor contributing to management and prognosis of type 2 diabetes is the stage of disease, which is possibly reflected by medication and degree of complications. We could have obtained information on medication use but the interpretation of the results are not straightforward. Medication use may both be a proxy of an advanced disease and a well-managed disease.

The participants in the Danish Diet, Cancer and Health cohort had higher socioeconomic position than non-participants [13] and this may impact the generalisability of the findings. The median plasma enterolactone concentration of 10.9 nmol/l (5th–95th percentile 1.3–59.6) was unexpectedly low compared with previous data from the same cohort (20 nmol/l [5th–95th percentile 3–90] for women and 18.6 nmol/l [5th–95th percentile 3–87] for men) [9]. In these earlier studies, the upper cut-off for the lowest enterolactone quartile was around 10 nmol/l, which is approximately the same as the upper limit of the second quartile in the present study. Since we used the same analytical method in the present study as in previously published studies on cancer cases from the same cohort [9], we were able to perform a validation on overlapping samples, confirming that type 2 diabetes cases had considerably low enterolactone concentrations compared with cancer cases from the same study population. A plausible explanation for this discrepancy could be the observed changes in the microbiota of people with diabetes, characterised by lower levels of clostridia [31] and bifidobacteria [32]. Both of these bacteria are involved in the deglycosylation step of the metabolism of lignans to enterolactone [28, 33]. This could possibly lead to problems of reverse causation, if the low enterolactone concentrations were caused by diabetes development-induced microbial changes. However, despite the low concentrations, we did observe an association between enterolactone and mortality. Another issue relates to the potential selection of people with low enterolactone concentrations into the study population. This is known as the ‘obesity paradox’ and is always relevant to consider in prognostic study designs [34]. One can speculate whether those being diagnosed with diabetes, despite high enterolactone concentrations, are genetically predisposed to developing a more aggressive type where lifestyle factors have less impact on progression. When we stratified on health status (BMI, smoking and physical activity) the associations were similar across health-status groups, weakening the concern that differences in lifestyle explain the observed association.

Risk factors related to mortality among people with type 2 diabetes in the USA include older age, male sex, lower income, smoking and higher LDL-cholesterol levels [3]. In a meta-analysis of intervention studies, both lignan-rich flaxseeds and lignan supplements were associated with reduced LDL-cholesterol level, possibly caused by increased enterolactone concentrations [5]. The most important dietary factors related to diabetes mortality include high intake of processed meats and sugar-sweetened beverages and low intake of whole grains [1]. In Denmark, whole grains are the most important source of plant lignans [35] and are generally linked to intake of dietary fibre and other associated phytochemicals [36] while smoking has previously been associated with lower enterolactone concentrations [37]. The significant association observed for diabetes-specific mortality is not likely explained by enterolactone itself, but rather indicates that people with type 2 diabetes dying from their disease as such generally have poorly managed diabetes and poor overall health reflected in low enterolactone concentrations. Our results suggest that people diagnosed with type 2 diabetes have better prognosis related to high enterolactone concentrations. High enterolactone concentrations can be obtained from intake of lignan-rich foods. In a dietary intervention with whole-grain rye (high lignan) vs refined wheat (low lignan), plasma enterolactone concentration was increased by 12 nmol/l following the high-lignan diet [38], a difference similar to that between quartile 1 and quartile 3 in the current study. Further, accumulating evidence supports the notion that dietary fibre elicits beneficial effects on variables of diabetes management [39], and high intakes of whole grains, fruit, vegetables, legumes and nuts (good sources of lignans) have been linked to improved glycaemic control and blood lipids in people with type 2 diabetes [40]. Taken together, this emphasises the importance of promoting a diet high in dietary fibre and lignans (e.g. including whole grains and vegetables) for the management of type 2 diabetes.

### Conclusion

Based on data from the Diet, Cancer and Health cohort study, pre-diagnostic enterolactone concentrations were inversely associated with all-cause and diabetes-specific mortality. Adjustment for antibiotic medication use did not attenuate the results and there were no signs of effect modification by health behaviour—although we still cannot rule out the possibility that enterolactone is a marker of general health. Seemingly, no published studies have so far reported on enterolactone and type 2 diabetes mortality. Therefore, our findings are novel but further exploration of the potential role of enterolactone in type 2 diabetes management is required.

## Electronic supplementary material

Table 1 (PDF 13 kb)

## Data Availability

The data that support the findings of this study are available from the Danish Cancer Research Center. Restrictions apply to the availability of these data, which were used under license for the current study and so are not publicly available. Data are however available from the authors upon reasonable request and with permission of the Regional Ethical Committees on human studies in Copenhagen and Aarhus and by the Danish Data Protection Agency.

## Abbreviations

DNPR: Danish National Prescription Registry
NHSR: Danish National Health Service Register
