# Supplementary material for: Pre-diagnostic plasma enterolactone concentrations are associated with lower mortality among individuals with type 2 diabetes: a case-cohort study in the Danish Diet, Cancer and Health cohort
Source: Diabetologia. 2019 Apr 8;62(6):959–69. doi: 10.1007/s00125-019-4854-9 (PMC6509069; doi:10.1007/s00125-019-4854-9)
Supplement: Supplementary file 1 — (PDF 13 kb) [file 125_2019_4854_MOESM1_ESM.pdf]

ESM Table 1.

Linear by doubling in concentration (log2)	Mortality					
	**All causes**	**Diabetes**	**Cardiovascular**	**Cancer**	**Respiratory**	**Other**
**n=596**	**n=274**	**n=21**	**n=51**	**n=130**	**n=23**	**n=42**
Model 2[b]	0.89 (0.81, 0.97)	0.64 (0.46, 0.88)	0.84 (0.68, 1.03)	0.92 (0.80, 1.05)	0.93 (0.76, 1.15)	0.93 (0.78, 1.11)
Model 2[c]	0.89 (0.81, 0.97)	0.62 (0.44, 0.87)	0.84 (0.68, 1.03)	0.92 (0.80, 1.05)	0.92 (0.74, 1.14)	0.93 (0.78, 1.10)
	Among non-users*					
**n=430**	**n=193**	**n=15**	**n=38**	**n=89**	**n=15**	**n=31**
Model 2[b]	0.89 (0.79, 1.00)	0.60 (0.38, 0.94)	0.88 (0.69, 1.13)	0.91 (0.78, 1.08)	0.93 (0.67, 1.29)	0.93 (0.75, 1.15)

[b] *adjusted for sex, age (5-year bands), smoking (never, former, current), BMI status (<27, 27-32, >32 kg/m2)*
[c] *adjusted for sex, age (5-year bands), smoking (never, former, current), BMI status (<27, 27-32, >32 kg/m2) + antibiotics: 0-3 months before baseline, 3-12 months before baseline or no use (12 months preceding baseline)*
** Sensitivity analyses of non-users of antibiotics (12 months before baseline) among persons with type 2 diabetes with information in the Danish National Prescription Registry the case-cohort study based on the Diet, Cancer and Health cohort (n=430)*

ESM Table 1. HR (95% CI) for pre-diagnostic plasma enterolactone concentrations and all-cause and cause-specific mortality among persons with type 2 diabetes with information in the Danish National Prescription Registry to adjust for antibiotic medication in the case-cohort study based on the Diet, Cancer and Health cohort (n=596)
